# Therapeutic effects of laparotomy and laparoscopic surgery on patients with gastric cancer

**DOI:** 10.12669/pjms.313.6528

**Published:** 2015

**Authors:** Yang Hu, Gaoping Zhao, Heng Zheng

**Affiliations:** 1 Yang Hu, Sichuan Academy of Medical Science & Sichuan Provincial People’s Hospital, Chengdu 610072, P. R. China; 2 Gaoping Zhao, Sichuan Academy of Medical Science & Sichuan Provincial People’s Hospital, Chengdu 610072, P. R. China; 3 Heng Zheng, Sichuan Academy of Medical Science & Sichuan Provincial People’s Hospital, Chengdu 610072, P. R. China

**Keywords:** Gastric cancer, Laparoscopic surgery, Laparotomy

## Abstract

**Objective:**

To compare the therapeutic effects of laparotomy and laparoscopic surgery on patients with gastric cancer.

**Methods:**

Sixty-six patients with gastric cancer who were treated in our hospital from January 2012 to December 2013 were selected and divided into a control group and an observation group by the random number method (n=33). The control group was treated by traditional laparotomy, and the observation group was treated by laparoscopic surgery. CD_4_/CD_8_ ratios and IgG expressions in the patients were detected on preoperative and postoperative fourth days. Intraoperative blood loss, surgical time, time of anal gas evacuation and time of postoperative independent ambulation of the two groups were observed.

**Results:**

The intraoperative blood loss, surgical time, time of anal gas evacuation, time of postoperative independent ambulation, time of urinary catheter indwelling and average hospitalization stay length of the observation group were significantly different from those of the control group (P<0.05). The postoperative rates of fever and complications in the observation group were significantly lower than those of the control group, and the two groups had significantly different CD_4_/CD_8_ ratios and IgG levels on the postoperative 4th day (P<0.05).

**Conclusion:**

Compared with traditional laparotomy, laparoscopic surgery can well treat patients with gastric cancer minimally invasively. Meanwhile, their postoperative recovery was facilitated due to slightly affected humoral immunity and cellular immune function.

## INTRODUCTION

Gastric cancer, as a common malignant tumor in the digestive system in clinical practice, has high mortality and morbidity rates.^1^ Recently, the incidence rate of gastric cancer has been increasing annually owing to the changes of lifestyle, dietary structure and pattern.^2^ Patients with gastric cancer are traditionally treated by laparotomy which, however, decelerates recovery and severely affects the postoperative quality of life because they are intrinsically different in physical status and age.^3^ Particularly, some patients may die of major surgical traumas.^4^ With the development of medical techniques, laparoscopy has been widely applied in clinical practice.

In this study, we aimed to improve the survival and quality of life of patients with gastric cancer by comparing the therapeutic effects of laparotomy and laparoscopic surgery.

## METHODS

### Baseline clinical data

Sixty-six patients with gastric cancer who were treated in our hospital from January 2012 to December 2013 were selected as the subjects. This study was approved by the Ethics Committee of our hospital. Informed consent was obtained from each patient and the study protocol conformed to the ethical guidelines of the 1975 Declaration of Helsinki. They were diagnosed as gastric cancer without distant metastasis by preoperative pathological examination. All patients were subjected to lymphatic metastasis after surgeries, and they were free from contraindications for surgeries. Then they were divided into a control group and an observation group by the random number method (n=33).

### Control group

23 males and 10 females; 42-76 years old (average: 62.0 ± 1.0); cancer staging: 13 cases of Stage I, 14 cases of Stage II and 6 cases of Stage III.

### Observation group

22 males and 11 females; 43-78 years old (average: 62.5 ± 1.0); cancer staging: 15 cases of Stage I, 13 cases of Stage II and 5 cases of Stage III. Their baseline clinical data were similar (P>0.05).

### Methods

### Control group

This group was treated by traditional laparotomy in the supine position under general intravenous anesthesia.^5^ The abdomen was cut open in the middle, 15-20cm in length surrounding the umbilicus. Afterwards, peritoneal metastases were explored to determine the surgical range. According to practical requirements, the greater omentum was separated, and the perigastric arteriovenous blood circulation was blocked. Meanwhile, lymph nodes were dissected before perioperative distal anastomosis and digestive tract reconstruction. The duodenum was disconnected, with the residual end cut by using an endoscopic stapler, and the pulled gastric tissues and greater omentum were closed. Finally, most of the omentum and distal stomach were resected extra-abdominally, and Billroth II gastrojejunal anastomosis and reconstruction were performed. After the surgery, a drainage tube was placed at the incision of right anterior axillary line. The two groups had the same range of surgical resection and requirements of lymphadenectomy.

### Observation group

This group was treated by laparoscopic surgery while retaining gastric tube and urinary catheter in the dorsal elevated position under general intravenous anesthesia. CO_2_ insufflation pressure was controlled at 1.6 kPa, and a 10mm Trocar was placed 1cm below the umbilicus, with a 30° mirror as the observation port. Two 5mm Trocars were placed bilaterally 2 cm outside the subcostal midclavicular line, and another 5mm Trocar was put 3cm below the midline umbilicus of left clavicle. Moreover, a 12mm Trocar was placed in the symmetrical position.^6^ Subsequently, exploration and lymph node dissection were conducted according to the procedure mentioned above for the control group. Finally, incisions of the abdominal cavity were sutured successively, and each puncture hole was sutured layeredly. Afterwards, the abdominal cavity was cleaned, and a drainage tube was placed.^7^ Antibiotics were routinely given after the surgery.

### Observation indices

CD_4_/CD_8_ ratios and IgG levels of the patients were detected on the fourth days before and after surgeries and compared. Intraoperative blood loss, surgical time, time of anal gas evacuation, time of postoperative independent ambulation, number of patients with postoperative fever, and postoperative complications of the two groups were observed. Peripheral venous bloods were sampled on the preoperative and postoperative 4th days (5ml each), and stored at -4°C after anticoagulation with heparin.^8^ The expressions of T lymphocyte subsets were detected by flow cytometry, and those of serum immunoglobulin were detected by double antibody sandwich ELISA.^9^

### Statistical analysis

All data were analyzed by SPSS19.0. Each index was expressed as mean ± standard deviation (*x̄* ± s), and the postoperative rates of complications and fever were expressed as %. All data were compared by χ² or t test, and P<0.05 was considered statistically significant.

## RESULTS

### Observation indices

The intraoperative blood loss, surgical time, time of anal gas evacuation, time of postoperative independent ambulation, time of urinary catheter indwelling and average hospitalization stay length of the observation group were (119.52 ± 6.24) ml, (65.21 ± 9.36) min, (0.65 ± 0.12) d, (3.21 ± 0.69) d, (0.59 ± 0.15) d and (12.52 ± 2.36) d respectively, which were significantly different from those of the control group [(351.21 ± 12.02) ml, (98.65 ± 10.21) min, (1.75 ± 0.36) d, (6.32 ± 0.75) d, (2.01 ± 0.27) d and (18.26 ± 3.07) d] (P<0.05) (Table-I).

**Table-I:** Postoperative observation indices (*x̄* ± s).

Index	Observation group (n=33)	Control group (n=33)	t	P
Intraoperative blood loss (ml)	119.52 ± 6.24	351.21 ± 12.02	9.571	<0.05
Surgical time (min)	65.21 ± 9.36	98.65 ± 10.21	6.462	<0.05
Time of anal gas evacuation (d)	0.65 ± 0.12	1.75 ± 0.36	16.975	<0.05
Time of postoperative independent ambulation (d)	3.21 ± 0.69	6.32 ± 0.75	7.623	<0.05
Time of urinary catheter indwelling (d)	0.59 ± 0.15	2.01 ± 0.27	3.871	<0.05
Average hospitalization stay length	12.52 ± 2.36	18.26 ± 3.07	12.736	<0.05

### Indices of immune function

The two groups had significantly different levels of CD_4_/CD_8_ and IgG on the postoperative 4th day (P<0.05) (Table-II).

**Table-II:** Indices of immune function before and after surgeries (*x̄* ± s).

Group	Time	IgG (g/L)	CD_4_/CD_8_
Control group (n=33)	Before	7.96 ± 2.29	1.91 ± 0.69
	After	13.89 ± 2.01	1.56 ± 0.58
t		3.852	7.452
P		<0.05	<0.05
Observation group (n=33)	Before	7.95 ± 2.27	1.89 ± 0.71
	After	8.12 ± 2.42	1.64 ± 0.59
t		1.642	4.785
P		>0.05	<0.05
ta		0.852	0.694
P		>0.05	>0.05
tb		3.659	3.211
P		<0.05	<0.05

### Complications

Observation group: Two cases of postoperative fever; complications: one case of postoperative infection and one case of small intestinal obstruction. Control group: six cases of postoperative fever; complications: Three case of postoperative infections, two case of small intestinal obstruction and one case of fat liquefaction at incision. The postoperative rates of fever and complications in the observation group (6.06% and 6.06% respectively) were significantly lower than those of the control group (18.18% and 18.18% respectively) (χ²=3.75, 7.88, P<0.05).

## DISCUSSION

With the rapid development and progress of modern medicine, laparoscopy has been widely applied in clinical practice.^10^ Compared with laparotomy, laparoscopic surgery is superior in minor traumas, quick recovery and mild stimulation. Since surgeries are bound to affect the immune system, the pros and cons of these two protocols were compared in this study by detecting postoperative immune indices.

The observation group had significantly less intraoperative blood loss, time of anal gas evacuation, time of urinary catheter indwelling, average hospitalization stay length, as well as incidence rates of fever and complications than the control group did.

Laparoscopy has now been given first priority among the patients with gastric cancer in our hospital because of minimal invasion, short hospitalization stay length and rapid recovery. Meanwhile, they enjoyed small abdominal scars and mild intra-abdominal adhesions.^11^ In 1994, Kitano et al., for the first time, performed laparoscopy-assisted Billroth I gastrectomy under abdominal wall elevation to treat early gastric cancer.^12^ Leung et al. reported that laparoscopy-assisted surgery decreased pain, postoperative incidence rate of complications and morality rate, also allowing rapid recovery and shortening the length of hospital stay.^13^ Kim et al. have also recommended laparoscopic gastrectomy as a standard protocol to treat early gastric cancer.^14^

Generally, long-term pneumoperitoneum is established during laparoscopic surgery, so the physical functions of patients may be affected,^15^ especially in their immune function. Hence, we herein compared the two surgical methods in regard to immune response. Of all the immune response products, IgG is of high level in human serum and extracellular fluid, which plays key roles in anti-infection and humoral immunity. The IgG level of the observation group remained almost unchanged after surgeries, suggesting that laparoscopic surgery barely affected the immune function. It has also been reported that the lgG levels of both laparoscopy-assisted and open radical gastrectomy groups decreased, but that of the latter group reduced more obviously, suggesting that laparoscopy-assisted gastrectomy exerted less inhibitory effects on systemic humoral immunity.^16^

Cell-mediated immunity is crucial in human immune response. After surgery, traumas reversely change the specific immune functions of patients, which are mainly manifested as damages to cell-mediated immunity, i.e. the changes of T lymphocytes. In human body, immunocompetent T lymphocytes mainly comprise CD_4_ and CD_8_ subgroups.^17^ Under normal conditions, the two subgroups are maintained dynamically balanced. Under trauma-induced stress, such balance is destroyed, thus altering the ratio of CD_4_/CD_8_.^18^ Both surgical methods decreased the CD_4_/CD_8_ ratio by bringing about damages, indicating that inhibitory subtype increased while auxiliary subtype reduced. On the postoperative 4th day, the observation group was significantly less prone to inflammatory reaction than the control group. Therefore, laparoscopic surgery was less traumatic.

In summary, laparoscopic surgery excelled traditional laparotomy both in physical and immune functions. In the meantime, the patients who received laparoscopic surgery recovered faster safely, with milder inflammatory reaction. Accordingly, this method, which is highly tolerant, is worthy of promotion and application in clinical practice. However, samples with larger sizes are in need, and more observation indices well be included in future studies.
